# Cannabidiol reverses attentional bias to cigarette cues in a human experimental model of tobacco withdrawal

**DOI:** 10.1111/add.14243

**Published:** 2018-06-03

**Authors:** Chandni Hindocha, Tom P. Freeman, Meryem Grabski, Jack B. Stroud, Holly Crudgington, Alan C. Davies, Ravi K. Das, William Lawn, Celia J. A. Morgan, H. Valerie Curran

**Affiliations:** ^1^ Clinical Psychopharmacology Unit University College London London UK; ^2^ National Addiction Centre, Institute of Psychiatry Psychology and Neuroscience, King's College London London UK; ^3^ School of Experimental Psychology University of Bristol Bristol UK; ^4^ Psychopharmacology and Addiction Research Centre University of Exeter Exeter UK

**Keywords:** Abstinence, attentional bias, cannabidiol, cigarette dependence, craving, withdrawal

## Abstract

**Background and Aims:**

Cannabidiol (CBD), a non‐intoxicating cannabinoid found in cannabis, may be a promising novel smoking cessation treatment due to its anxiolytic properties, minimal side effects and research showing that it may modify drug cue salience. We used an experimental medicine approach with dependent cigarette smokers to investigate if (1) overnight nicotine abstinence, compared with satiety, will produce greater attentional bias (AB), higher pleasantness ratings of cigarette‐related stimuli and increased craving and withdrawal; and (2) CBD in comparison to placebo, would attenuate AB, pleasantness of cigarette‐related stimuli, craving and withdrawal and not produce any side effects.

**Design:**

Randomized, double‐blind cross‐over study with a fixed satiated session followed by two overnight abstinent sessions.

**Setting:**

UK laboratory.

**Participants:**

Thirty non‐treatment‐seeking, dependent cigarette smokers recruited from the community.

**Intervention and comparator:**

800 mg oral CBD, or matched placebo (PBO) in a counterbalanced order

**Measurements:**

AB to pictorial tobacco cues was recorded using a visual probe task and an explicit rating task. Withdrawal, craving, side effects, heart rate and blood pressure were assessed repeatedly.

**Findings:**

When participants received PBO, tobacco abstinence increased AB (*P* = 0.001, *d* = 0.789) compared with satiety. However, CBD reversed this effect, such that automatic AB was directed away from cigarette cues (*P* = 0.007, *d* = 0.704) and no longer differed from satiety (*P =* 0.82). Compared with PBO, CBD also reduced explicit pleasantness of cigarette images (*P =* 0.011; *d =* 0.514). Craving (Bayes factor = 7.08) and withdrawal (Bayes factor = 6.95) were unaffected by CBD, but greater in abstinence compared with satiety. Systolic blood pressure decreased under CBD during abstinence.

**Conclusions:**

A single 800‐mg oral dose of cannabidiol reduced the salience and pleasantness of cigarette cues, compared with placebo, after overnight cigarette abstinence in dependent smokers. Cannabidiol did not influence tobacco craving or withdrawal or any subjectively rated side effects.

## Introduction

More than 1.1 billion people smoke world‐wide 1. A primary addictive driver of cigarette smoking is nicotine withdrawal. Withdrawal occurs upon cessation and includes physiological symptoms (headaches, nausea), affective symptoms (anxiety, depression and irritability) and impaired cognitive performance (delay discounting, response inhibition) 2, which peak within the first few days 3. Some evidence suggests withdrawal severity predicts relapse 3, 4, 5, 6, prevention of which is a major challenge in the treatment of addiction 7. Even when using the currently most effective smoking cessation drug (varenicline), a majority still fail to maintain long‐term abstinence 8. Nicotinic medications may also have unpleasant side effects, e.g. nausea 9.

There is mounting evidence that the endogenous cannabinoid (eCB) system is involved in motivation for rewards, including modulating the rewarding effects of drugs 10, 11, 12, 13, 14, 15. In relation to nicotine dependence, cannabinoid receptor 1 (CB1R) antagonists, such as rimonabant, decrease nicotine conditioned place preference and self‐administration in pre‐clinical models of addiction 16, 17. In human clinical trials, rimonabant increased smoking abstinence rates 1.6‐fold 18, 19. Although potentially effective, rimonabant was withdrawn from the market due to serious neuropsychological side effects.

Cannabidiol (CBD) is the second most abundant cannabinoid in cannabis. It has been shown to have broad therapeutic benefits 20, 21 and is showing initial promise as a treatment for addiction, anxiety and schizophrenia. The psychological properties of CBD are suggestive of a potentially ideal drug for smoking cessation. These include its lack of intoxicating and subjective effects 22, 23, 24, alongside its anxiolytic 25, 26 effects in humans. Its anxiolytic properties are particularly relevant, as anxiety is a primary symptom of tobacco withdrawal 27. The first human pilot study to investigate CBD as a treatment for nicotine dependence randomized participants to either 1 week of *ad‐hoc* CBD or placebo inhaler to be used when they had the urge to smoke. CBD reduced the number of cigarettes reportedly smoked by almost 40%, in comparison to placebo, but did not affect craving 28. No neurocognitive mechanisms through which CBD may assist with the treatment of smoking cessation were investigated. On the basis of previous findings 29, the authors proposed that a reduction in the salience of drug cues could be one candidate mechanism.

Attentional bias is a potentially important in‐laboratory predictive marker of the salience of drug cues. It is heightened, as indexed by dot‐probe tasks, during acute abstinence 2; predicts short‐term relapse 30; and is thought to play a causal role in maintaining addiction 31. Attentional bias to tobacco stimuli at a short (compared to longer) exposure interval is particularly important, as tobacco abstainers show greater bias to these cues only at short exposure 32. CBD may reduce the salience of smoking cues, which would be consistent with pre‐clinical, human experimental and neuroimaging research. In human naturalistic research, cannabis with high, in comparison to low, levels of CBD reduced cue salience to cannabis‐related stimuli in a visual probe task 29. This was again only observed at the short stimulus exposure interval which taps ‘automatic’ bias, i.e. that which is not subject to conscious cognitive control. As such, CBD may target an important implicit process involved in relapse. In a pre‐clinical rat model of addiction, Ren *et al*. 33 showed that CBD (5–20 mg/kg) attenuated cue‐induced heroin‐seeking behaviour and relapse, which was maintained for 2 weeks after CBD administration. Furthermore, human translational pilot research showed that a single dose of CBD can attenuate cue‐induced craving in heroin users during a 24‐hour period and this was maintained for 7 days 34. One neuroimaging study suggests that CBD modulates activity of areas in the brain associated highly with salience attribution, including the striatum, hippocampus and prefrontal cortex 35. Taken together, the experimental evidence provides a strong rationale to hypothesize that CBD is a potential treatment for substance use disorders where the salience of drug cues is key.

This is the first study, to our knowledge, to investigate the effects of CBD during nicotine withdrawal in humans. We employ an experimental medicine approach to investigate CBD's potential to target processes relevant to smoking cessation. Human laboratory studies of smoking abstinence provide an efficient, cost‐effective, mechanistic evaluation of medications for smoking behaviour 36, which may facilitate translational research. Specifically, we hypothesized that: (1) overnight nicotine abstinence, compared with satiety, will produce a range of nicotine withdrawal symptoms in dependent cigarette smokers which include greater attentional bias (short stimulus exposure), higher pleasantness of cigarette‐related stimuli and increased craving and withdrawal; (2) CBD in comparison to placebo, would attenuate attentional bias and pleasantness of cigarette‐related stimuli, craving and withdrawal symptomology relative to pre‐drug scores; and (3) CBD in comparison to placebo, will not produce any significant cardiovascular or side effects.

## Material and methods

### Design and participants

Thirty participants attended three sessions [mean = 7.85, standard deviation (SD) = 2.77 days between sessions]. Participants smoked as normal before their first (baseline) session, verified with expired carbon monoxide (CO) ≥ 10 parts per million (p.p.m.) (Bedfont Scientific, Harrietsham, UK). Participants then attended two sessions after overnight (~12‐hour) abstinence, verified by CO ≤ 10 p.p.m. 37. A double‐blind, placebo‐controlled, cross‐over design was used to compare the effects of 800 mg oral CBD with matched placebo (PBO) after overnight smoking abstinence. Treatment order for abstinent sessions was randomized and counterbalanced. Participants received the drug based on a randomization code, balanced for gender (http://www.random.org), which was concealed from experimenters until all data were collected and entered. Drug concealment occurred through participant‐numbered, opaque, sealed envelopes. There was a minimum washout period of 1 week between drug sessions to preclude potential CBD carry‐over effects following previous research 23, 24.

Dependent cigarette smokers were recruited from the community through on‐line message boards. Inclusion criteria were: (i) age 18–50 years; (ii) smoking ≥ 10 cigarettes a day for at least the last year; (iii) Fagerström Test for Nicotine Dependence (FTND) score ≥ 4 (moderate dependence) 38; (iv) smoking first cigarette within an hour of waking; and (iv) negative drug urine screen for all major drugs of abuse at baseline. Exclusion criteria were: (i) use of nicotine replacement therapy/cessation pharmacotherapy; (ii) self‐reported recent use of cannabis or other illicit drugs; (iii) recent (past 4 weeks) or ongoing use of e‐cigarettes; (iv) current mental or physical health issues or learning impairments; (v) pregnancy or breastfeeding; and (vi) allergies to CBD, gelatine, lactose, microcrystalline cellulose or chocolate.

### Power calculation

We calculated that *n* = 20 would be necessary to have power of 95% at an alpha of 5% to detect a large effect size of *d* = 0.78 (*F* = 0.38). This was based on the difference in the number of cigarettes smoked pre–post 1 week of CBD inhaler versus placebo (23.25 cigarettes) in Morgan *et al*. 28. This sample size was increased by 50%, yielding a final sample of 30 to adjust for ‘winner's curse’ 39, i.e. over‐inflation of effect sizes from initial positive studies.

### Drug administration

Participants were administered 800 mg oral CBD [pure synthetic (−) CBD, STI Pharmaceuticals, Brentwood, UK] or matched placebo (lactose powder) in identical, opaque capsules on each testing occasion. 800 mg was chosen, as it produces an increase in plasma concentrations after acute administration [C_max_ = 77.9, standard error = 25 ng/ml, T_max_ = 180 minutes 23), is well tolerated in humans, is efficacious for schizophrenia 40, increases extracellular anandamide levels 40 and should be sufficient to influence salience attribution after a single dose 35.

### Assessments

#### Visual probe task (Fig. 1)

This task was implemented as a measure of attentional bias 41. Thirty smoking (target) and composition‐matched neutral (non‐target) images were shown 42. Each trial began with a fixation point (500 ms). A pair of images then appeared on the left and right of the screen for either a short (200 ms) or long (500 ms) duration to assess automatic orientating and controlled attention processing, respectively. Image pairs were replaced by a probe (an arrow pointing upwards or downwards) in the location of either the neutral or smoking‐related image. The probe remained on screen until the participant responded to identify the probe orientation (upwards or downwards) by pressing one of two appropriate response keys as quickly and accurately as possible (defined as a ‘correct trial’ if a correct response was made). Probes replaced the cigarette‐related and neutral images equally often. The position of image type, probe location and stimulus duration was counterbalanced. Trials were displayed in a single block with each pair presented eight times, producing 80 critical trials and 32 neutral trials. The task began with four buffer trials. Trial order was randomized each time the task was run. The task was programmed with Experiment Builder (SR Research, Kanata, ON, Canada).

**Figure 1:**
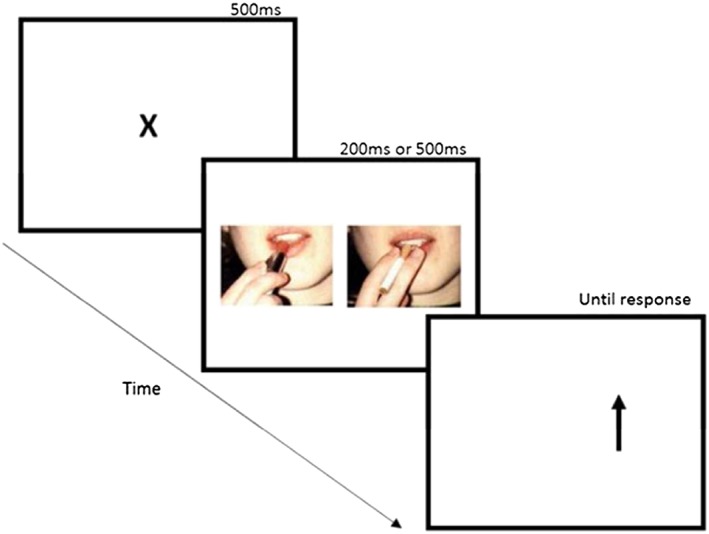
Trial structure for the visual probe task. Example of cigarette (right) and matched neutral stimuli (left) provided. [Colour figure can be viewed at http://wileyonlinelibrary.com]

#### Pleasantness rating task (PRT)

Each trial began with a fixation cross of 500 ms, followed by either a cigarette or neutral cue, presented in a randomized order for 3000 ms. Stimuli were matched on brightness and complexity. Cigarette stimuli involved smoking‐related scenes and were the same as the visual probe. Participants rated the pleasantness of each image on a scale of −3 (very unpleasant) to +3 (very pleasant). Valence was recorded. Three versions were available for counterbalancing. The experiment was conducted using Psychopy 43, 44.

### State questionnaires

Withdrawal was assessed with the Mood and Physical Symptoms Scale (MPSS) 45. Craving was assessed with Questionnaire of Smoking Urges–Brief (QSU‐B) 46. Participants completed a six‐item side‐effect form with items: ‘strong drug effect’, ‘good drug effect’, ‘willing to take drug again’, ‘like drug effect’, ‘I have an upset stomach’ and ‘I have a headache’. Each item was rated on a 10‐point visual analogue scale (VAS) from ‘not at all’ to ‘extremely’.

### Trait questionnaires

The FTND was used to assess nicotine dependence 38. Anxiety was assessed with the State–Trait Anxiety Inventory (STAI) 47 and depression with the Beck Depression Inventory (BDI) 48. A comprehensive drug history was taken 49. Pre‐morbid verbal intelligence was indexed by the Spot the Word task 50.

### Procedure

After telephone screening eligible participants attended a baseline ‘satiated session’, prior to which they smoked as normal. This involved further screening assessments (CO, urine test, pregnancy test, Spot the Word) as well as the same assessments as on the abstinent days. On the satiated day, participants completed state measures of craving (QSU‐B) and withdrawal (MPSS) after they were deemed eligible (T1; +12 minutes), were asked to smoke a cigarette (Marlboro Gold) to ensure satiety (+30 minutes), then completed a second measure of craving and withdrawal (T2; + 35 minutes), the visual probe task (+ 60 minutes), PRT (+68 minutes) and a final measure of craving and withdrawal (T3; +75 minutes). On abstinent sessions, participants attended two ~ 3.5‐hour sessions, separated by 1 week, after overnight abstinence. They provided a CO reading, then completed state questionnaires and cardiovascular measures [QSU‐B, MPSS, heart rate (HR), blood pressure (BP) (T1, +5 minutes)]. CBD or matched placebo was then administered orally (+10 minutes). After drug administration, participants completed half the trait questionnaires in each session. At 70 minutes (T2) and 130 (T3) minutes they again completed the MPSS, QSU‐B, HR and BP. Participants then completed the visual probe (+180 minutes) and PRT (+188 minutes). At 200 minutes, participants completed a final measure of craving and withdrawal (T4). A detailed schedule of assessments can be found in Supporting information, Table S1. Other assessments are reported elsewhere 51. All participants provided written informed consent. Ethical approval was given by UCL Ethics Committee. Participants were reimbursed £10 per hour.

### Statistical analysis

Statistical analyses were performed in the Statistical Package for Social Scientists (SPSS version 24; IBM, Chicago, IL, USA). Visual inspection of diagnostic plots was used to check for normality. Where the assumption of sphericity was violated, the Greenhouse–Geisser correction was used and rounded to the nearest integer; η^2^p denotes partial eta‐squared. Outliers > 1.5 × the interquartile range (IQR) were Winsorized to the next highest/lowest value. For the PRT, 4.2% of the data were missing due to technical issues and were replaced with the means of the condition. Sensitivity analysis showed that Winsorization or mean imputation did not modify any result.

Only correct trials (99.97% of the data) were analysed for the visual probe and responses > 2000 and < 200 ms were removed. Following Mogg *et al*. 52, bias scores were calculated for the visual probe and PRT, such that a positive score indicates a bias towards cigarette cues. This was calculated as the difference in RT between when the probe replaced the neutral, in comparison to cigarette, stimulus (RT_neutral_ – RT_cigarette_) for the visual probe task; and as cigarette_valence – neutral_valence for the PRT.

The visual probe and PRT were analysed using repeated‐measures analysis of variance (ANOVA) with two *a priori* orthogonal Helmert contrasts to investigate main effects. The first describes the main effect of abstinence, i.e. satiated (SAT) versus abstinent (CBD). The second describes the main effect of drug, i.e. CBD versus PBO. For the visual probe task, an additional task‐specific factor of exposure time was included to investigate automatic (short) in comparison to strategic (long) processing. Interactions between condition and exposure were explored via pairwise *post‐hoc* comparisons, Bonferroni‐corrected locally within each omnibus term.

Craving (QSU) and withdrawal (MPSS) symptomology were analysed with two repeated‐measures ANOVAs because of the difference in timing between sessions and number of assessments of craving and withdrawal. The first investigated satiation (T2: immediately after a cigarette) versus abstinence (T1: pre‐drug administration). The second compared CBD in comparison to PBO across all time‐points [T1 (pre‐drug), T2, T3, T4]. Interactions between condition and time were assessed with *post‐hoc* comparisons, Bonferroni‐corrected locally within each omnibus term.

Side effects, HR and BP were measured three times on abstinent sessions, therefore these data were analysed with a 2 [CBD, PBO) × 3 T1 (pre‐drug), T2, T3] ANOVA. Interactions between condition and time were assessed with *post‐hoc* comparisons, Bonferroni‐corrected locally within each omnibus term.

Scaled Jeffreys–Zellner–Siow (JZS) Bayes factors (BF) were calculated when the main effect of drug (CBD versus PBO) was not significant according to frequentist statistics (*P* > 0.05). We used a scaled‐information prior of *r* = 1 53.

Carry‐over effects were assessed using an additional between‐subjects factor of ‘order’. No order effects were found for the main analyses (as evidenced by no interactions or main effects involving treatment order). Therefore, we report results without accounting for order. As we did not have any specific a priori hypotheses regarding covariates, we did not include any, as per Kraemer 54.

## Results

### Participant characteristics (Tables 1 & 2)

Thirty participants (14 female) took part. The sample had a mean (SD) age of 28.07 (8.66) years, with an FTND score of 5.56 (1.13) demonstrating moderate dependence. They smoked 13.5 (2.39) cigarettes per day, which is slightly more than the national adult average of 11.5 55. Further demographics, trait scores and cigarette smoking information can be found in Table 1. Use of other drugs was minimal in this population (Table 2). For confirmation of both self‐reported and CO level indexed abstinence; see Supporting information.

**Table 1:** Participants’ demographic and trait variables. Results are displayed as mean (standard deviation).

n	30
Age (years)	28.07 (8.66)
FTND score	5.56 (1.13) range 4–8
Cigarettes per day	13.5 (2.39) range 10–20
Time to first cigarette (mins)	25.5 (15.87)
Years smoked	9.55 (7.36)
Years smoking > 10+ cigarettes/day	8.17 (7.08)
Life‐time quit attempts (*n* = 25)	3.2 (3.91)
Most successful quit attempt (days)	100.48 (163.47)
Body mass index	23.98 (7.78)
Spot the Word	48.03 (4.15)
STAI	40.53 (9.4)
BDI	10.36 (7.54)

FTND = Fagerström Test for Nicotine Dependence; STAI = Stait–Trait Inventory; BDI = Beck Depression Inventory.

**Table 2:** Drug use history (n = the number of people who used the drug in the past year). Results are displayed as mean (standard deviation).

	Alcohol	Cannabis	MDMA	Cocaine
*n*	26	17	9	9
Days since last use	6.39 (10.13)	100 (68.30)	84.66 (82.22)	100 (56.12)
Number of years used	13.08 (8.68)	8.29 (4.61)	4.55 (1.59)	3.33 (2.12)
Days per month	11.43 (8.85)	0.75 (1.30)	0.67 (1.32)	0.5 (1.15)
Typical amount per session	7.1 units (3.23)	0.87 joints (0.69)	258.33 mg (144.70)	800 mg (0.83)

MDMA = 3,4‐methylenedioxymethamphetamine.

### Attentional bias

#### Visual probe task (Fig. 2)

**Figure 2:**
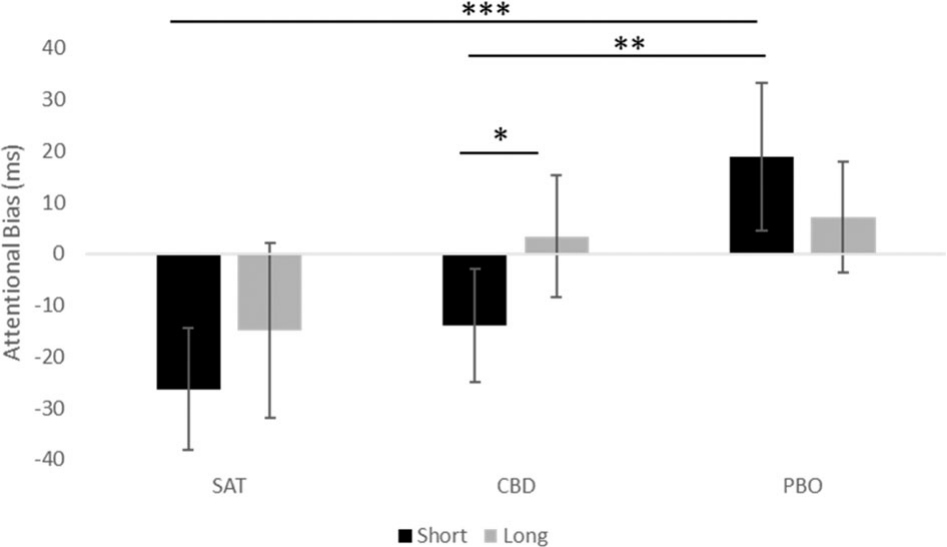
Attentional bias across satiated (30 min post‐cigarette) and abstinent (180 min post‐drug administration) for both short and long exposure times. Estimated marginal means are presented with 95% confidence interval error bars. *P ≤ 0.05; **P ≤ 0.01; ***P ≤ 0.001. SAT=satiated; CBD=cannabidiol; PBO=placebo

There was a main effect of abstinence (*F*_(1,29)_ = 9.52, *P* = 0.004, η^2^p = 0.27), which showed that there was a greater attentional bias under abstinence versus satiation. There was a main effect of drug, which was subsumed under the condition × exposure interaction (*F*_(2,58)_ = 4.66, *P* = 0.013, η^2^p = 0.14). The interaction showed that under the short stimulus exposure, there was greater attentional bias to cigarette cues in the PBO condition, in comparison to SAT (45.15 ms, 95% CI = 71.77, 18.54, *P* = 0.001, *d* = 0.789), as well as greater attentional bias in the PBO condition in comparison to CBD (36.47 ms, 95% CI = 64.18, 8.77, *P* = 0.007, *d* = 0.704), but not between SAT and CBD (−8.68 ms, 95% CI = –28.43, 11.07, *P =* 0.82). Under the long stimulus exposure, none of these comparisons were significant. Additionally, AB was greater to cigarette cues under the long, in comparison to short, exposure time for CBD (20.94 ms, 95% CI = 40.29, 5.15, *P* = 0.015), but not under SAT (*P* = 0.263) or PBO (*P* = 0.155). There was no main effect of exposure time (*F*_(1,29)_ = 2.14, *P* = 0.155, η^2^p = 0.07).

### Pleasantness rating task

#### Valence (Fig. 3)

**Figure 3:**
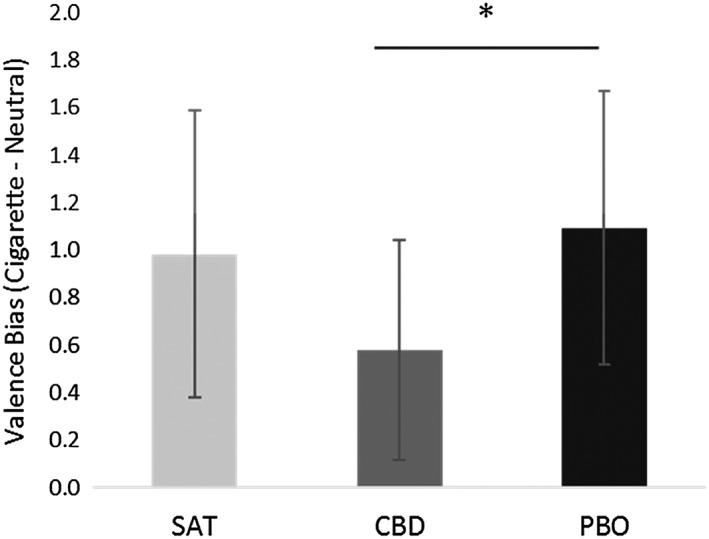
Bias in pleasantness rating (calculated as cigarette valence minus neutral valence) for satiated (38 min post‐cigarette) and abstinent (188 min post‐drug administration) conditions. Estimated marginal means are presented with 95% confidence interval error bars. *P ≤ 0.05

There was no main effect of abstinence (*F*_(1,29)_ = 0.53, *P* = 0.47, η^2^p = 0.02). There was a significant main effect of drug (*F*_(1,29)_ = 7.41, *P* = 0.011, η^2^p = 0.20), indicating less bias towards cigarette stimuli on CBD compared to PBO (−0.51, 95% CI = –0.99, −0.03); *d =* 0.514).

### Craving (Fig. 4)

**Figure 4:**
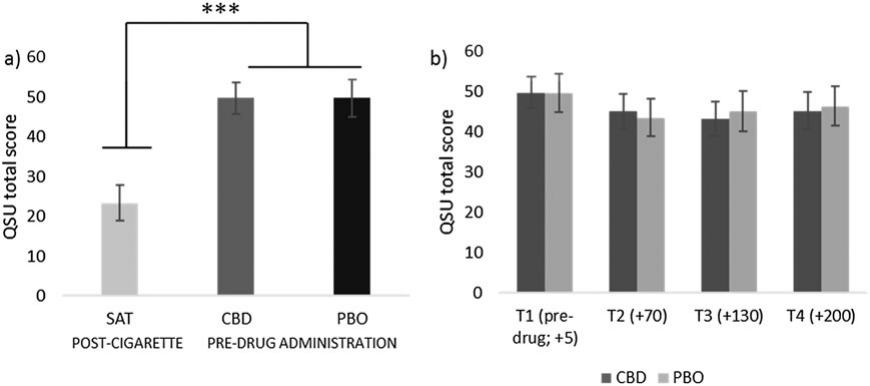
Scores for the Questionnaire of Smoking Urges–Brief (QSU‐B) (craving). Left panel (a) shows significantly greater craving on abstinent sessions before drug administration, in comparison to satiation scores after a cigarette. Right panel (b) compares cannabidiol (CBD) and matched placebo (PBO) across all time‐points pre‐ and post‐drug administration (T2 onwards). See Supporting information, Table S1 for details on timing. Estimated marginal means with 95% confidence interval are presented. ***P ≤ 0.001

Pre‐drug QSU scores were greater in abstinent conditions versus satiation (*F*_(1,29)_ = 99.75, *P* < 0.001, η^2^p = 0.78). There was no difference between CBD and PBO, pre‐drug administration (*P =* 0.99) confirmed by a Bayesian analysis, showing that the null was 7.08 more likely than the alternative given the data (JZS BF = 7.08). To investigate if CBD attenuated craving in comparison to PBO on abstinent sessions, we conducted an ANOVA that showed a main effect of time (*F*_(2,54)_ = 8.34, *P* < 0.001 η^2^p = 0.22); however, there was no main effect of drug (*P* = 0.81) confirmed by a Bayesian analysis (JZS BF = 6.87) or drug × time interaction, suggesting no difference between CBD and PBO.

### Withdrawal (Fig. 5)

**Figure 5:**
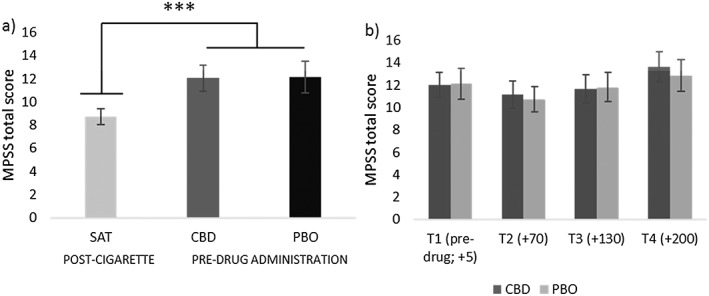
Scores for the Mood and Physical Symptoms Scale (MPSS) (withdrawal symptoms). Left panel (a) shows significantly greater withdrawal on abstinent sessions before drug administration, in comparison to satiation scores after a cigarette. Right panel (b) compares cannabidiol (CBD) and matched placebo (PBO) across all time points pre‐ and post‐drug administration (T2 onwards). See Supporting information, Table S1 for details on timing. Estimated marginal means with 95% confidence interval are presented. ***P ≤ 0.001

Pre‐drug MPSS scores was greater under abstinent conditions versus satiation (*F*_(1,29)_ = 29.88, *P* < 0.001, η^2^p = 0.51), suggesting that abstinence increased withdrawal. There was no difference between CBD and PBO, pre‐drug administration (*P =* 0.85) confirmed by Bayesian analysis showing the null was 6.95 more likely than the alternative given the data (JZS BF = 6.95). To investigate if CBD attenuated withdrawal in comparison to PBO on abstinent sessions, we conducted an ANOVA that showed a main effect of time (*F*_(2,69)_ = 8.98, *P <* 0.001, η^2^p = 0.24); however, there was no effect of drug (*F*_(1,29)_ = 0.22, *P =* 0.64, η^2^p = 0.01) confirmed by a Bayesian analysis (JZS BF = 6.35) or drug × time interaction.

Analysis of the additional MPSS questions (amount of time spent with urges and strength of urges) can be found in Supporting information.

### Cardiovascular effects

#### Heart rate (HR)

There was a main effect of time (*F*_(1,39)_ = 33.73, *P* < 0.001, η^2^p = 0.54) which showed that HR decreased over time. There was no main effect of drug (*P* = 0.30) confirmed by a Bayesian analysis (JZS BF = 4.17) and no interaction between drug and time.

#### Blood pressure (BP)

A main effect of drug (*F*_(1,29)_ = 6.72, *P* = 0.015, η^2^p = 0.19) showed higher systolic BP after PBO than after CBD (+3.40, 95% CI = 0.72–6.08). There was a main effect of time (*F*_(2,58)_ = 13.24, *P* < 0.001, η^2^p = 0.31), which showed that systolic BP decreased over time. There were no main effects or interactions for diastolic BP.

### Side effects

One interaction between drug and time was found for ‘headache’, but no significant pairwise comparisons emerged. No other main effects of drug or interactions were found between drug and time. See Supporting information for more details.

## Discussion

This study employed an experimental medicine approach to investigate the effects of a single 800‐mg oral dose of CBD on nicotine withdrawal. We found evidence that, compared to placebo, CBD reversed attentional bias to cigarette cues in abstinent smokers, such that it was no longer significantly different from attentional bias when they were satiated. Simultaneously, we observed a reduction in explicit pleasantness during abstinence, such that cigarette stimuli were rated as less pleasant after CBD than placebo. These neurocognitive effects occurred in the absence of any changes in subjective states of craving and withdrawal between CBD and placebo. This suggests that CBD may have specific effects on the evaluative and motivational salience‐reducing properties of drug cues, which is consistent with clinical 29, 34 and pre‐clinical research 33. Moreover, no significant psychoactive or side effects were observed. These results therefore support the potential of CBD in targeting specific neurocognitive processes in nicotine addiction.

To be specific, a reduction in the implicit salience of drug cues of a large effect size was observed in the CBD condition (versus placebo) after overnight abstinence in dependent cigarette smokers. That is to say that participants were over 40 ms faster to detect probes replacing smoking (versus neutral) cues under placebo than under CBD. This was observed in the short exposure time only, consistent with our initial hypothesis and with previous findings regarding attentional bias 32 and CBD 29. The short exposure time is related to implicit automatic processing and initial orientation to cues, which occur outside the individual's explicit awareness 32, 56.

These results suggest that one potential candidate mechanism by which CBD may exert anti‐addictive effects is by normalizing the salience of drug cues. This in line with the incentive salience model of drug addiction 57. Given that attentional bias may predict smoking cessation outcomes 30, CBD may be useful in aiding early abstinence by reducing the salience of drug‐related cues. However, it is unlikely that attentional bias is the only driver of nicotine addiction, and other mechanisms require investigation.

As well as effects of CBD on implicit attentional bias, a reduction in explicit pleasantness for cigarettes under CBD compared to placebo was also observed. Explicit pleasantness is important with regard to addiction because it partly indexes the reinforcing value of a drug. In humans, users of high, in comparison to low CBD : THC ratio cannabis showed lower self‐reported pleasantness of cannabis stimuli, which follows the same pattern as the present study 29 and may be related to endocannabinoid involvement in hedonic experiences 58. However, there was no difference between abstinence and satiated sessions, which was unexpected, as it was hypothesized and has been shown previously 59.

The absence of CBD effects on withdrawal and craving are surprising because, theoretically, the incentive salience model of Robinson & Berridge 57 would suggest that a reduction in attentional bias would be accompanied by a reduction in craving. Moreover, Hurd *et al*. 34 found that CBD reduced cue‐induced craving and anxiety which was maintained for 24 hours in heroin users (however, a different paradigm was used). It is notable that both Morgan *et al*. 28 and the present study did not find effects on tonic craving, therefore CBD may not be effective for all smokers but only those suffering from heightened attentional bias to drug cues. The incentive salience model equates craving with wanting a drug, not liking a drug, and argues that craving reflects the attribution of intense incentive salience to reward‐associated stimuli. In the present research, CBD reduced attentional bias, arguably an index of incentive salience, but had no impact on craving. Given that craving and attentional bias are dissociated here, with CBD specifically attenuating attentional bias, this research seems to be inconsistent with the model. It may be the observed reduction in attentional bias is a result of a general motivational effect in that CBD may be reducing general orienting to salient cues, thus explaining the observed dissociation. Future research should investigate whether CBD also modifies orientating to other salient stimuli such as food cues. This has been investigated with street cannabis, where individuals smoking cannabis high (in comparison to low) in CBD had significantly lower attentional bias to both cannabis and food‐related cues 29.

The neurobiological mechanism by which CBD may exert these effects is unclear; however, a promising candidate is through normalization of extracellular anandamide, via inhibition of fatty acid amide hydrolase (FAAH). FAAH inhibitors have been shown to reduce nicotine self‐administration and conditioned place preference (CPP) in rats and monkeys as well as nicotine‐induced dopamine release in the nucleus accumbens 60, 61, 62, 63. Here, we were unable to measure anandamide levels; however, this putative mechanism requires further research, as more potent FAAH inhibitors may provide more anti‐addictive effects than CBD. This may also be the mechanism by which CBD may alleviate psychotic symptoms in people with schizophrenia 40.

### Limitations

First, we used an experimental medicine approach to investigate mechanistic effects of single‐dose CBD during overnight tobacco withdrawal, therefore it is unclear whether these effects will translate to the clinic and how long they might last. The visual probe task provides only a cross‐sectional snapshot of attentional bias in a laboratory setting, and may suffer from low internal reliability 64. In this case, ecological momentary assessment may be more indicative of attentional bias in actual drug‐taking environments. Additionally, use of eye tracking, functional magnetic resonance imaging (fMRI) or electroencephalogram (EEG) would provide additional information on the time–course and neural correlates of attentional bias. Moreover, only a single dose of CBD was given; future research needs to investigate repeated dosing and a range of doses 65. Finally, compliance with tobacco smoking abstinence instructions was verified with breath CO, but abstinence from other nicotine products was based on self‐report, therefore we could not verify objectively that participants had not used other nicotine products. However, craving and withdrawal scores were markedly higher under abstinence than satiation, suggesting that self‐report was reliable.

## Conclusions

This is the first study, to our knowledge, to investigate effects of CBD on nicotine withdrawal. After overnight tobacco abstinence, cigarette smokers administered 800 mg CBD, in comparison to placebo, show a reduced salience and pleasantness of cigarette cues, in the absence of any reductions in withdrawal or craving. This study highlights the potential utility of CBD as a treatment for specific neurocognitive components of tobacco use disorder, and suggests that one potential mechanism by which CBD may exert its effects on addiction is via a reduction in the salience of drug cues. These results support the growing literature regarding CBD in the treatment of addictive disorders.

## Declaration of interests

None.

## Supporting information


**Figure S1** Flow diagram for study recruitment and assessments. The final sample included 30 participants who completed all three sessions.
**Table S1** Schedule of assessments on the satiated and abstinent sessions.
