# Supplementary material for: Cannabidiol reverses attentional bias to cigarette cues in a human experimental model of tobacco withdrawal
Source: Addiction. 2018 Jun 3;113(9):1696–705. doi: 10.1111/add.14243 (PMC6099309; doi:10.1111/add.14243)
Supplement: Supplementary file 1 — Figure S1 Flow diagram for study recruitment and assessments. The final sample included 30 participants who completed all three sessions. Table S1 Schedule of assessments on the satiated and abstinent sessions. [file ADD-113-1696-s001.docx]

# SUPPLEMENTARY MATERIALS

**Cannabidiol reverses attentional bias to cigarette cues in a human experimental model of tobacco withdrawal.**

Hindocha, C^1*^., Freeman, T.P^1,2^., Grabski, M^1,3^., Stroud, J.B^1^., Crudgington, H^1^., Davies, A.C.^1^., Das, R.K^1^., Lawn, W^1^., Morgan, C.J.A^1,4^., Curran, H.V.^1^

^1^Clinical Psychopharmacology Unit, University College London, WC1E 7HB

^2^National Addiction Centre, Institute of Psychiatry, Psychology and Neuroscience, King’s College London, London, SE5 8BB, United Kingdom.

^3^School of Experimental Psychology, University of Bristol, 12a Priory Road, BS81TU, Bristol.

^4^Psychopharmacology and Addiction Research Centre, University of Exeter, UK

**Running Head:** CANNABIDIOL FOR TOBACCO WITHDRAWAL

**Correspondence to:** Chandni Hindocha, Clinical Psychopharmacology Unit, University College London, 1-19 Torrington Place, London, WC1E 7HB. Email: [c.hindocha@ucl.ac.uk](mailto:c.hindocha@ucl.ac.uk)

# Supplementary Method

## Participant recruitment


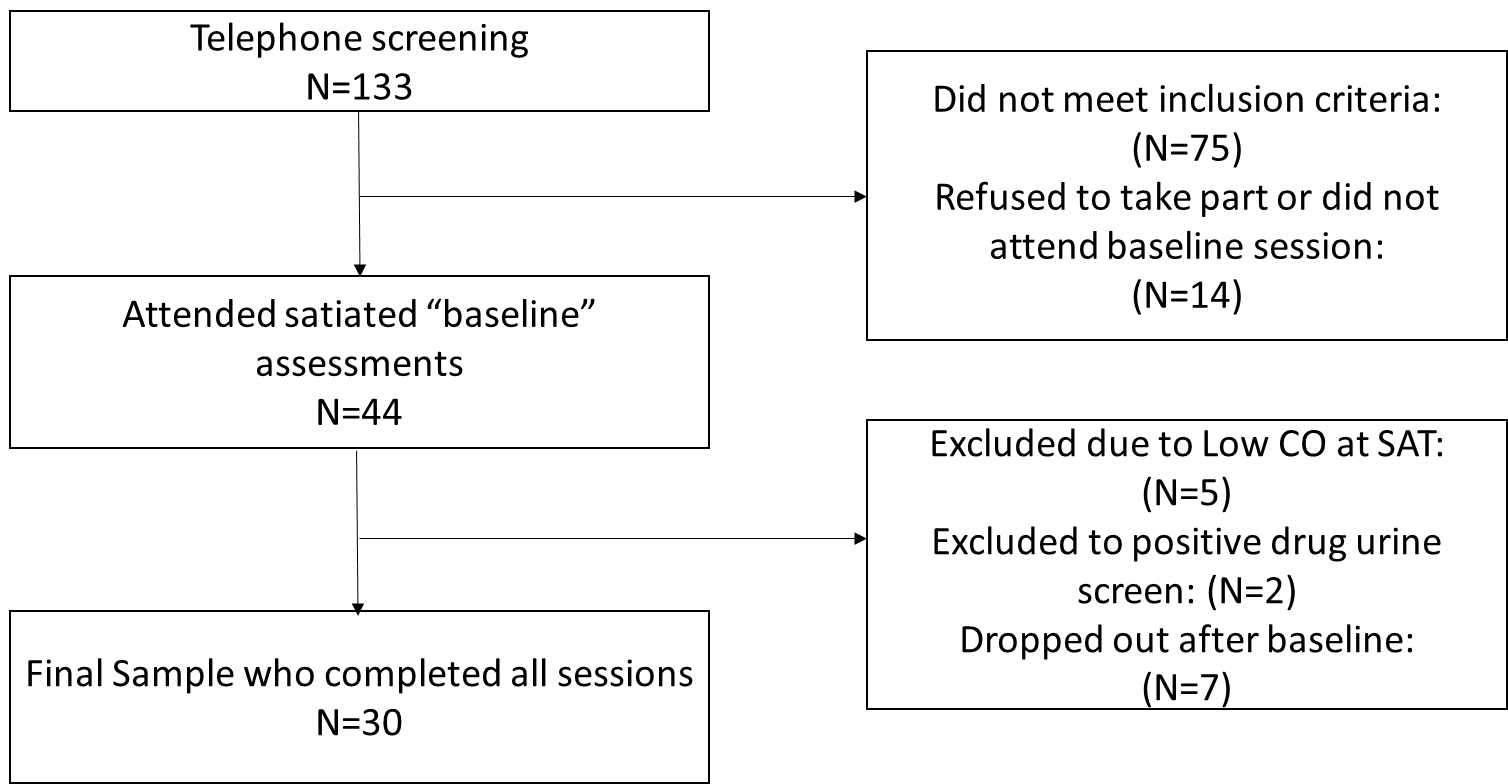


*Supplementary Figure 1: flow diagram for study recruitment and assessments. The final sample included 30 participants who completed all three sessions.*

## Procedure

*Supplementary table 1: Schedule of assessments on the satiated and abstinent sessions.*

| **SATIATED** | | **ABSTINENT** | |
| --- | --- | --- | --- |
| **TIME** |  | **TIME** |  |
| **0** | Arrival | **0** | Arrival |
| **12** | MPSS QSU [1] | **5** | MPSS QSU HR BP [1] |
| **30** | Cigarette | **10** | Drug administration |
| **35** | MPSS QSU [2] | **70** | MPSS QSU HR BP [2] |
| **60** | Visual Probe | **130** | MPSS QSU HR BP [3] |
| **68** | PRT | **190** | Visual Probe |
| **75** | MPSS QSU [3] | **198** | PRT |
| - | - | **200** | MPSS QSU [4] |

# Supplementary Results

## Time since last smoked

There was a significant main effect of abstinence (*F*(1,29)= 3289.03, *p*<.001, *η*^2^*p* =.99) where on the satiated session, participants last smoked M: 0.41 (SD: 0.40) hours previously, in comparison to abstinent. There was no main effect of drug (*F*(1,29)=0.18, *p*=.675, *η*^2^*p*=.006). Participants last smoked M: 10.97 (SD:0.96) hours previously on the CBD session and M:11.03 (SD:0.95) on the PBO session.

## CO

There was a significant main effect of abstinence (*F*(1,29)= 167.83 *p*<.001, *η*^2^*p*=.84) which shows CO was higher in the satiated condition (M: 17.73 ppm SD: 6.63) than in the abstinent conditions. There was no main effect of drug (*F*(1,29)=6.13, *p*=.019, *η*^2^*p*=.17) where CO was 4.27ppm (SD:2.23) for CBD and 4.17 (SD:2.69) for PBO. Thus abstinence was biologically verified.

# MPSS

## Amount of time spent with urge

Pre-drug time spent with urges was significantly greater under abstinent than satiated sessions *F*(1,29)=27.96, *p*<.001, *η*^2^*p*=.49 suggesting abstinence increased the amount of time spent with urges to smoke. There was no different between CBD and PBO, pre-drug administration (*p*=0.536; JZS BF in support of the null= 5.86). To investigate if CBD attenuated craving in comparison to placebo on abstinent sessions, we conducted an ANOVA that showed a main effect of time (*F*(3,87)=8.65, *p<*.001, *η*^2^*p*=.23) which showed that time spent with urges decreased from T1 (3.17, 95% CI 2.79-3.64) to T3 (2.40, 95% CI 1.97-2.82), and increased from T3 to T4 (2.80, 95% CI 2.38-3.22). However there was no effect of drug (*p*=1.00; JZS BF in support of the null= 7.08) There was no drug x time interaction *F*(2, 68)=.25, *p=*.81, *η*^2^*p*=0.00).

## Strength of urges

Pre-drug strength of urges was significantly greater under abstinent than satiated sessions *F*(1,29)=26.26, *p*<.001, *η*^2^*p*=.48 suggesting abstinence increased the strength of urges. There was no different between CBD and PBO, pre drug administration (*p*=0.879; JZS BF in support of the null= 6.99). To investigate if CBD attenuated craving in comparison to placebo on abstinent sessions, we conducted an ANOVA that showed a main effect of time (*F*(3,87)=4.33, *p=*.007, *η*^2^*p*=.13) which showed that time spent with urges decreased significantly from T1 (2.92, 95% CI 2.58-3.25) to T2 (2.40, 95% CI 2.02-2.78), and increased from T2 to T3 (2.48, 95% CI 2.10-2.87) and T4 (2.73, 95% CI 2.31-3.16). However there was no effect of drug (*p=*.61; JZS BF in support of the null= 6.20) There was no drug x time interaction *F*(3, 87)=0.65, *p=*0.58, *η*^2^*p*=0.02).

# Side effects

*Strong Drug effect:* There was no main effect of drug (*F*(1,29)=.80, *p*=.379, *η*^2^*p*=.03) confirmed by Bayesian analysis (JZS BF:  4.82), time (*F*(2,58)=.37 *p*=.695, *η*^2^*p* =.01), or drug x time interaction (*F*(2,58)=2.18, *p=*.123,  *η*^2^*p*=.07).

*Good Drug effect:* There was no main effect of drug (*F*(1,29)=.10, *p*=.922, *η*^2^*p*=.00) confirmed by Bayesian analysis (JZS BF:7.04), time (*F*(2,58)=2.76, *p*=.072,  *η*^2^*p* =.09), or drug x time interaction (*F*(2,58)=2.18, *p=*.123, *η*^2^*p* =.07).

*Willing to take drug again:* There was no main effect of drug (*F*(1,29)=2.35, *p*=.136, *η*^2^*p*=.08) confirmed by Bayesian analysis (JZS BF: 2.35), time (*F*(2,58)=0.42, *p*=.661, *η*^2^*p*=.01, or drug x time interaction (*F*(2,58)=1.12, *p=*.306, *η*^2^*p*=.040).

*Like drug effect:* There was no main effect of drug (*F*(1,29)=.01, *p*=.947, *η*^2^*p*=.00) confirmed by Bayesian analysis (JZS BF: 7.06) or drug x time interaction (*F*(2,58)=.03, *p=*.968, *η*^2^*p*=.00). There was a main effect of time (*F*(2,58)=3.53, *p*=.036, *η*^2^*p*=.11) which showed liking decreased over time.

*I have a stomach ache:* There was no main effect of drug (*F*(1,29)=.00, *p*=.957,  *η*^2^*p*=.00) confirmed by Bayesian analysis (JZS BF:7.07), time (*F*(2,58)=.01, *p*=.988, *ηp^2^*=.000), or drug x time interaction (*F*(2,58)=1.44, *p=*.245, *η*^2^*p*=.05).

*I have a headache:* There was a drug x time interaction (*F*(2,58)=3.17, *p=*.049,  *η*^2^*p*=.099). Exploration of the interaction showed no significant pairwise comparisons. There was no main effect of drug (*F*(1,29)=.04, *p*=.839, *η*^2^*p*=.00) confirmed by Bayesian analysis (JZS BF:6.93), or time (*F*(2,58)=.80, *p*=.456, *η*^2^*p*=.03).
